# Visibility of early gastric cancers by texture and color enhancement imaging using a high-definition ultrathin transnasal endoscope

**DOI:** 10.1038/s41598-023-29284-7

**Published:** 2023-02-03

**Authors:** Yohei Koyama, Mitsushige Sugimoto, Takashi Kawai, Midori Mizumachi, Fumito Yamanishi, Sho Matsumoto, Yuka Suzuki, Daiki Nemoto, Hirokazu Shinohara, Tadashi Ichimiya, Takahiro Muramatsu, Yasuyuki Kagawa, Taisuke Matsumoto, Akira Madarame, Takashi Morise, Kumiko Uchida, Hayato Yamaguchi, Shin Kono, Sakiko Naito, Masakatsu Fukuzawa, Takao Itoi

**Affiliations:** 1 Department of Gastroenterology and Hepatology, Tokyo Medical University Hospital, 6-7-1 Nishishinjuku, Shinjuku-ku, Tokyo, 160-0023 Japan; 2 Department of Gastroenterological Endoscopy, Tokyo Medical University Hospital, Tokyo, Japan

**Keywords:** Gastrointestinal cancer, Stomach diseases

## Abstract

We evaluated whether texture and color enhancement imaging (TXI) using a high-definition ultrathin transnasal endoscope (UTE) improves the visibility of early gastric cancer (EGC) compared with white-light imaging (WLI). This study included 31 EGCs observed by TXI mode 2 using a high-definition UTE prior to endoscopic submucosal dissection. The first outcome was to compare the color differences based on Commission Internationale de l’Eclairage L*a*b* color space between EGCs and the surrounding mucosa by WLI and TXI using the UTE (objective appearance of EGC). The second outcome was to assess the visibility of EGCs by WLI and TXI using the UTE in an image evaluation test performed on 10 endoscopists (subjective appearance of EGC). Color differences between EGCs and non-neoplastic mucosa were significantly higher in TXI than in WLI in all EGCs (TXI: 16.0 ± 10.1 vs. WLI: 10.2 ± 5.5 [mean ± standard deviation], *P* < 0.001). Median visibility scores evaluated by 10 endoscopists using TXI were significantly higher than those evaluated using WLI (TXI: 4 [interquartile range, 4–4] vs. WLI: 4 [interquartile range, 3–4], *P* < 0.001). TXI using high-definition UTE improved both objective and subjective visibility of EGCs compared with WLI.

## Introduction

Screening esophagogastroduodenoscopy (EGD) in health check-ups is widely known as an effective method to reduce the gastric cancer mortality rate by its early detection, particularly in East Asian countries with a high prevalence of *Helicobacter pylori* infection and gastric cancer^1,2^. Although white-light imaging (WLI) is currently used for screening endoscopy to detect early gastric cancer (EGC), the detection of EGC has remained difficult and relies on the expertise of the endoscopists^3,4^. Image-enhanced endoscopy (IEE) techniques, such as narrow-band imaging (NBI), blue-laser imaging, and linked color imaging have been reported to improve the detection rate of EGC compared with WLI^5–7^. In fact, the MAPS II guidelines, which are the official guidelines of the European Society of Gastrointestinal Endoscopy, state that high-definition endoscopy with IEE is more effective than high definition WLI alone for the diagnosis of EGC. The guidelines also state that IEE, with or without magnification, should be used for the diagnosis of gastric precancerous conditions, in guiding biopsies for staging atrophic and metaplastic changes, and to help target neoplastic lesions^8^. However, because even IEE cannot detect all EGCs, it is considered necessary to develop more efficient endoscopic technologies (e.g., artificial intelligence, high-vision endoscopy, and new processors) and advanced IEE systems.

A recently developed new processor is now available for use with the novel IEE, namely, texture and color enhancement imaging (TXI)^9–11^. There are two modes of TXI: TXI mode 2 automatically optimizes the “structure” and “brightness”, and TXI mode 1 also enhances the “color” of the gastrointestinal mucosa. TXI might therefore overcome the disadvantages of previously established diagnostic methods using WLI in screening EGD. Thus, TXI has the possibility of increasing the detection rate of EGC in daily clinical practice.

In Japan, the ultrathin transnasal endoscope (UTE) is widely used for screening EGD in clinics and health check-up institutions, owing to its safety and high acceptability from patients^12–14^. Previous first- and second-generation UTEs had low resolution, and it was hence difficult to distinguish various structures and contrasts of color between the EGC and the background gastric mucosa. However, the recently developed high-definition third-generation UTE has improved resolution, and it is becoming possible to detect EGCs and to make accurate endoscopic diagnoses using not only WLI but also IEE^15–19^. Nevertheless, the gold standard for screening EGD is still WLI. Therefore, TXI mode 2 without color enhancement, which provides similar images to WLI, might be useful for screening EGD using the currently available diagnostic methods. However, it is unclear whether the combination of the EVIS XI system, which is a new processor with improved image quality compared with older processors, such as EXERA III and LUCERA ELITE, and the third-generation UTE is useful for the detection of EGC by TXI mode 2 compared with WLI. Therefore, the aim of this study was to compare the visibility of EGCs between WLI and TXI mode 2, used together with the novel high-definition UTE and the EVIS XI system.

## Methods

### Study design and participants

**Figure 1:**
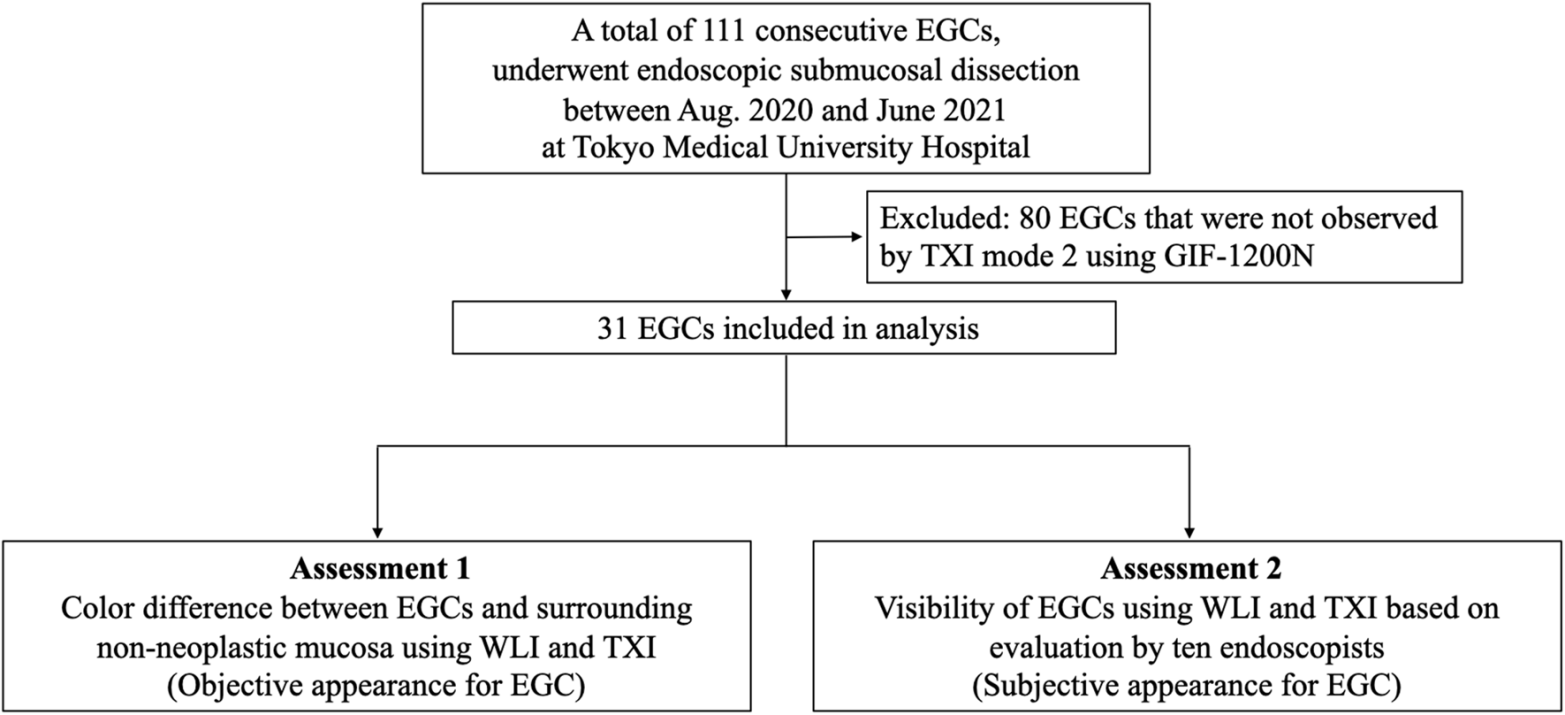
Study flowchart. *EGC* Early gastric cancer, *ESD* Endoscopic submucosal dissection, *TXI* Texture and color enhancement imaging, *WLI* White-light imaging.

A retrospective observational study was conducted to investigate the visibility of EGCs by TXI mode 2 using the high-definition third-generation UTE, GIF-1200N, and the EVIS XI system (Olympus Co., Tokyo, Japan) at a single tertiary center (Tokyo Medical University Hospital) in Japan. A total of 111 consecutive EGC patients who underwent endoscopic submucosal dissection (ESD) from August 2020 to June 2021 were initially analyzed (Fig. 1). Patients in whom TXI mode 2 observation using GIF-1200N was not performed were excluded.

This study was approved by the institutional review board of Tokyo Medical University Hospital (registration number: T2020-0380). The study protocol conforms to the ethical guidelines of the Declaration of Helsinki, as reflected by its a priori approval by the institution’s human research committee. Because this study was a retrospective observational study and written informed consent was not obtained from each patient, a document declaring an opt-out policy, through which any patient could refuse to be included in the study, was uploaded on the website of Tokyo Medical University Hospital.

### Endoscopic and pathological evaluations

EGC patients who were evaluated using the GIF-1200N scope and the EVIS XI system were retrospectively selected. The TXI settings were fixed at mode 2, which automatically adjusts the structure and brightness of the WLI. EGCs were routinely evaluated in the distant view, middle view, and near view using both WLI and TXI in all EGC patients before ESD. Pathological evaluations of EGCs were described in accordance with the criteria proposed by the Japanese Gastric Cancer Association^20^.

### Colorimetric evaluation of EGCs using WLI and TXI

**Figure 2:**
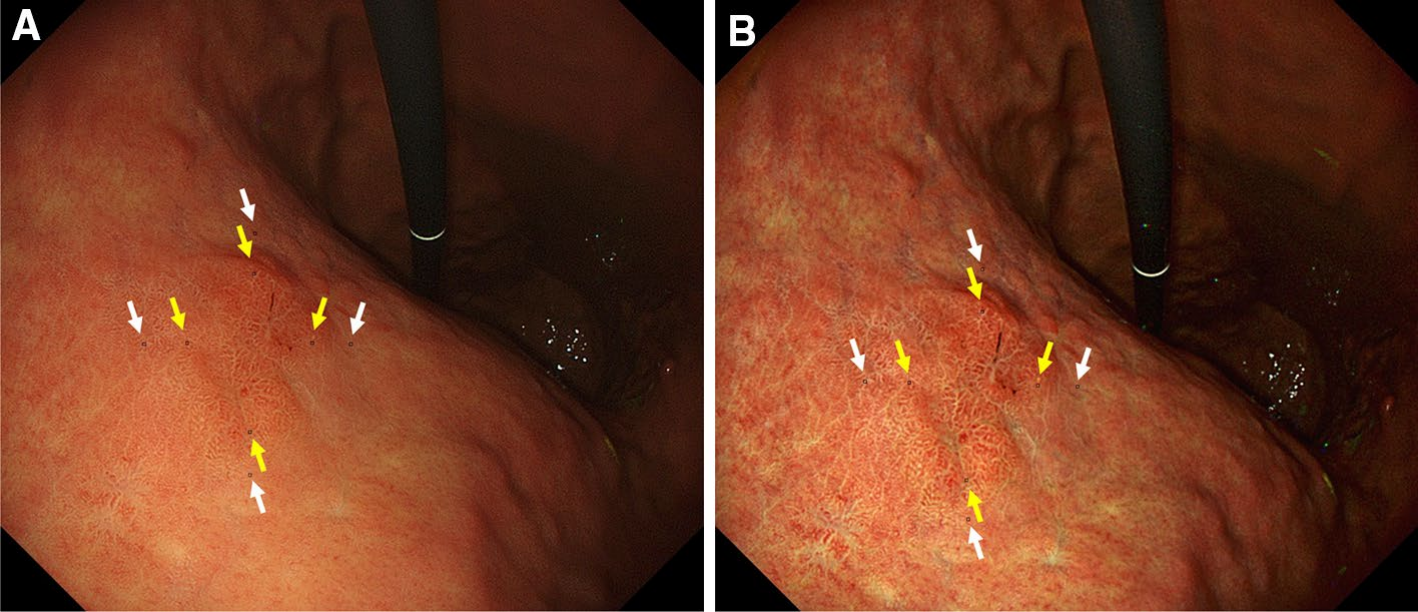
Examples of actual endoscopic images used in the colorimetric evaluation process. A reddish depressed lesion is seen in the posterior wall of the middle gastric body. A total of 8 ROI were annotated on the surrounding non-neoplastic mucosa and EGC. ROIs were set at the same point on the WLI (**A**) and TXI (**B**). The yellow arrows indicate the EGC, and the white arrows indicate the surrounding non-neoplastic mucosa. All ROIs were standardized to 25 pixels (5 × 5).

To evaluate the objective appearance of the EGCs by WLI and TXI using the high-definition UTE, color differences between EGC and the surrounding mucosa in the WLI and TXI images were assessed using Commission Internationale de l’Eclairage (CIE) L*a*b* color space^21,22^. This is a 3-dimensional space for presenting a color with axes of L* (from black to white; white is highest), a* (from green to red; red is highest), and b* (from blue to yellow; yellow is highest). A color difference was defined as ΔE, which expresses the distance between 2 points in the color space, and was calculated using the following formula: ΔE = [(ΔL*)2 + (Δa*)2 + (Δb*)2]^1/2^, in which ΔL*, Δa*, Δb* are differences in the L*, a*, and b* values, respectively. Initially, an expert endoscopist (T.K.) familiar with UTE retrospectively evaluated all the still images of the 31 EGCs, while being blinded regarding both the histopathological results and clinical information, and selected representative WLI and TXI images from the same site in the middle view. Then, a total of 8 regions of interest (ROI) were set for each endoscopic image. Four ROIs were set in the surrounding non-neoplastic mucosa, 2 mm outside the EGCs (proximal, distal, anterior, and posterior sides). The other 4 ROIs were set 2 mm inside the EGCs, corresponding to each direction of ROIs of the surrounding non-neoplastic mucosa also in the proximal, distal, anterior, and posterior sides (Fig. 2). In each lesion, ROIs were set at the same point on the WLI and TXI images. All ROIs were standardized to 25 pixels (5 × 5). Finally, L*a*b* inside the respective ROIs was measured, and the color contrast between EGCs and the surrounding mucosa was calculated from the mean value of ΔE.

### Visibility assessment of EGCs observed by WLI and TXI

To assess the subjective appearance of EGCs by WLI and TXI, an image evaluation test was performed by 10 endoscopists (5 experts and 5 trainees). Expert endoscopists were defined as those who were certified as endoscopists by the Japan Endoscopic Society. All other endoscopists were defined as trainees. The endoscopic images were selected from middle to distant views obtained under almost the same condition by an expert endoscopist (Y.K.) who was not an evaluator in the image evaluation test, while being blinded regarding both the histopathological results and clinical information. The 62 endoscopic images (31 WLI and 31 TXI) were randomly ordered and displayed, and the evaluators independently assessed them in a single session. The endoscopists scored the lesions based on a 4-point visibility scale, while being blinded regarding both the histopathological diagnosis and clinical information for each EGC^23,24^.

Visibility scores were defined as follows: 4, excellent visibility (easily detectable); 3, good visibility (detectable with careful observation); 2, fair visibility (hardly detectable without careful examination); and 1, poor visibility (not detectable without repeated careful examination).

### Assessment of H. pylori status

*H. pylori* infection was determined by an anti-*H. pylori* IgG serological test or a 13C-urea breath test (UBT). The absence of *H. pylori* after *H. pylori* eradication therapy was confirmed using a UBT. Patients who obtained negative results in both tests and had no endoscopic gastric atrophy or intestinal metaplasia were diagnosed as *H. pylori*-negative.

### Study outcomes

The first outcome was to compare the mean color differences between EGCs and the surrounding mucosa by WLI and TXI using the high-definition UTE (objective appearance for EGCs). The second outcome was to assess the visibility of EGCs by WLI and TXI using the high-definition UTE (subjective appearance for EGCs). The objective and subjective evaluations included comparison of *H. pylori*-positive and *H. pylori*-eradicated patients, and comparison of the elevated type and flat/depressed type of EGC. In addition, differences between experienced and trainee endoscopists were also assessed in the subjective evaluation.

### Statistical analysis

Parameters, including age, tumor diameter, and ΔE were expressed as the mean ± standard deviation (SD). The 2-tailed paired *t*-test was used to compare ΔE values. Visibility score was expressed as the median with interquartile range (IQR), and was compared using the Wilcoxon test. L*a*b* color measurements were performed using Adobe Photoshop 2022 (Adobe System, San Jose, CA, USA). The color differences using WLI and TXI were calculated using the CORREL function in Microsoft Excel 2019 (Microsoft, Redmond WA, USA). Statistical analyses were performed using SPSS version 28 software (IBM Corp, Armonk, NY, USA). A *P*-value of less than 0.05 was considered to indicate a statistically significant difference between groups.

### Ethics approval and consent to participate

All procedures involving human participants were performed in accordance with the ethical standards of our institution and/or national research committees, and with the 1964 Declaration of Helsinki and its later amendments or comparable ethical standards. We conducted this study in accordance with the guidelines of our institutional review board, which approved this retrospective study without the need for informed consent (Tokyo Medical University Hospital registration number T2020-0380).

## Results

### Clinicopathological characteristics of the EGC patients

Of the 111 consecutive EGC patients who underwent ESD from August 2020 to June 2021, 80 patients were not observed using TXI mode 2 and the GIF-1200N endoscope. None of the patients refused to be included in this study. Finally, a total of 31 EGC patients were enrolled in this study (Fig. 1).

The clinicopathological characteristics of the EGC patients are shown in Table 1. The mean age of the patients was 73.2 ± 8.7 years, and 77.4% of the patients were men. The mean tumor diameter was 12.1 ± 7.6 mm. Two-thirds of the tumors were located in the middle part of the stomach (n = 20, 64.5%), and were observed as having a reddish color (n = 20, 64.5%). The macroscopic tumor types were 13 elevated, 4 flat, and 14 depressed types. Regarding the pathological diagnoses, most of the EGCs were of the differentiated type (n = 30, 96.8%) and the invasion depth was pT1a (n = 30, 96.8%). *H. pylori* status of the patients was 7 positive, 21 eradicated, 2 negative, and 1 undetermined.

**Table 1:** Characteristics of the patients with EGC.

		(n = 31)
Age, years	Mean ± SD	73.2 ± 8.7
Sex	Male/female (n)	24/7
Tumor diameter, mm	Mean ± SD	12.1 ± 7.6
Location	Upper/middle/lower stomach (n)	3/20/8
Color of tumor in WLI	Reddish/discolored/isochromatic (n)	20/5/6
Macroscopic type	Elevated/flat/depressed (n)	13/4/14
Histological type	Differentiated/undifferentiated (n)	30/1
Tumor depth of invasion	T1a/T1b (n)	30/1
*H. pylori* status	Positive/eradicated/negative/undetermined (n)	7/21/2/1

*SD* Standard deviation, *WLI* White-light imaging.

### Color differences between EGCs and the surrounding mucosa by WLI and TXI

The mean ± SD color difference between EGCs and non-neoplastic mucosa was significantly higher in TXI than in WLI in all patients (16.0 ± 10.1 vs. 10.2 ± 5.5, *P* < 0.001) (Table 2). The difference was significantly higher in TXI regardless of whether the patients were *H. pylori*-positive (15.7 ± 9.3 vs. 10.9 ± 6.4, *P* < 0.001) or *H. pylori*-eradicated (16.2 ± 10.4 vs. 10.2 ± 5.2, *P* < 0.001). Regarding macroscopic type, the difference was significantly higher in TXI both for elevated lesions (16.3 ± 10.7 vs. 9.1 ± 5.5, P < 0.001) and flat/depressed lesions (15.8 ± 9.7 vs. 11.0 ± 5.7, *P* < 0.001) (Table 2).

**Table 2:** Color differences between EGCs and the surrounding mucosa on WLI and TXI (objective appearance of EGCs).

	WLI	TXI	*P*-value
All patients, n = 31	10.2 ± 5.5	16.0 ± 10.1	< 0.001
***H. pylori* status**			
Positive, n = 7	10.9 ± 6.4	15.7 ± 9.3	0.028
Eradicated, n = 21	10.2 ± 5.2	16.2 ± 10.4	< 0.001
**Macroscopic type**			
Elevated, n = 13	9.1 ± 5.0	16.3 ± 10.7	< 0.001
Flat or depressed, n = 18	11.0 ± 5.7	15.8 ± 9.7	< 0.001

Data regarding color differences are presented as the mean and standard deviation.

*TXI* Texture and color enhancement imaging, *WLI* white-light imaging.

Three patients, including 2 *H. pylori-*negative and 1 undetermined patient, were excluded from the analysis.

**Figure 3:**
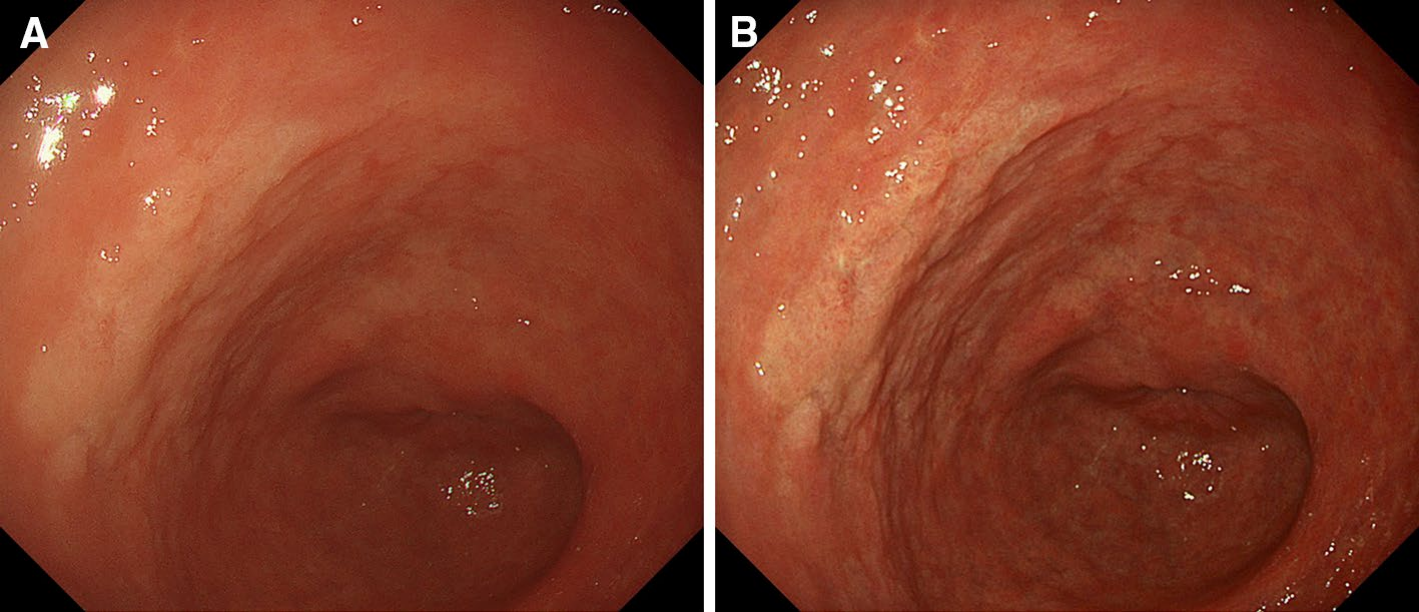
A representative case showing improved color differences on TXI. (**A**) WLI displayed a slightly discolored, flat elevated lesion in the anterior wall of the lower gastric body. The demarcation line is slightly difficult to identify. The mean ΔE was 8.5 ± 1.6. (**B**) TXI displayed contrast enhancement between the lesion and the surrounding mucosa, resulting in an easily recognizable demarcation line. The mean ΔE was 12.7 ± 4.3.

A representative case of EGC (*H. pylori-*eradicated patient with differentiated type adenocarcinoma, invasion depth pT1a) with improved color difference by TXI compared with WLI is shown in Fig. 3 (TXI: 8.5 ± 1.6, and WLI: 12.7 ± 4.3).

### Subjective visibility of EGCs by WLI and TXI

The median [IQR] of the visibility scores evaluated by 10 endoscopists were significantly higher for TXI than for WLI (TXI: 4 [4–4] vs. WLI: 4 [3–4], *P* < 0.001). This was similar for both experts (TXI: 4 [4–4] vs. WLI: 4 [3–4], *P* < 0.001) and trainees (TXI: 4 [4–4] vs. WLI: 4 [3–4], *P* < 0.001). In *H. pylori*-positive patients, there was no significant difference in visibility by TXI and WLI (TXI: 4 [4–4] vs. WLI: 4 [3–4], *P* = 0.154). On the other hand, subjective visibility was significantly higher by TXI (TXI: 4 [4–4] vs. WLI: 4 [3–4], *P* < 0.001) in *H. pylori*-eradicated patients. Regarding macroscopic type, visibility was significantly higher by TXI for both elevated TXI: 4 [4–4] vs. WLI: 4 [3–4], *P* = 0.013) and flat/depressed lesions (TXI: 4 [4–4] vs. WLI: 4 [3–4], *P* < 0.001) (Table 3).

**Table 3:** Visibility scores of EGCs using WLI and TXI (subjective appearance of EGCs).

	WLI	TXI	*P*-value
Total: n = 310 (31 × 10)	4 (3–4)	4 (4–4)	< 0.001
**Experience of endoscopist**			
Expert, n = 155 (31 × 5)	4 (3–4)	4 (4–4)	< 0.001
Trainee, n = 155 (31 × 5)	4 (3–4)	4 (4–4)	< 0.001
***Helicobacter pylori***			
Positive, n = 70 (7 × 10)	4 (3–4)	4 (4–4)	0.154
Eradicated, n = 210 (21 × 10)	4 (3–4)	4 (4–4)	< 0.001
**Macroscopic type**			
Elevated, n = 130 (13 × 10)	4 (3–4)	4 (4–4)	0.013
Flat or depressed, n = 180 (18 × 10)	4 (3–4)	4 (4–4)	< 0.001

Data on visibility scores are presented as the median and interquartile range.

*TXI* Texture and color enhancement imaging, *WLI* White-light imaging.

**Figure 4:**
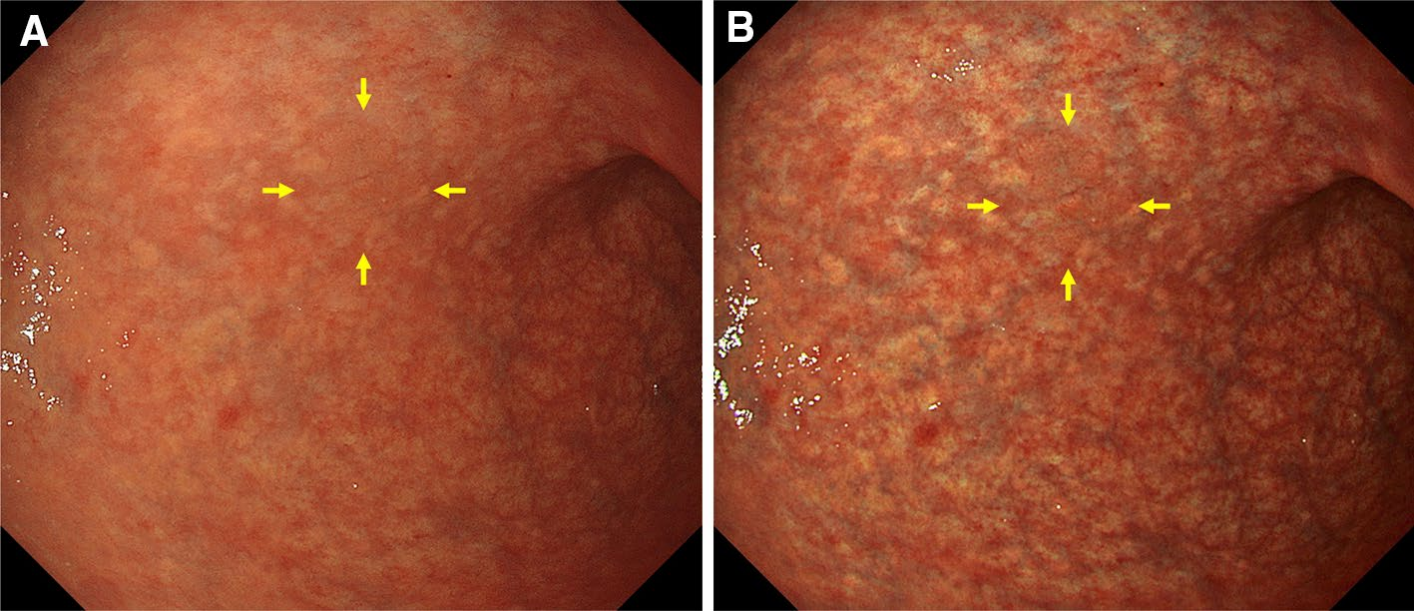
A representative case showing improved visibility on TXI. (**A**)WLI displayed an isochromatic flat lesion in the anterior wall of the lower gastric body. Although there is a slight difference in surface structure, it is difficult to recognize. The median (IQR) visibility by 10 endoscopists was 2 (1–4). (**B**) TXI displayed enhancement of the structure of the EGC, and the mucosal atrophy in the background appeared more whitish. The median (IQR) visibility by 10 endoscopists was improved to 3 (2–4). The yellow arrows in (**A**) and (**B**) indicate the EGC.

A representative case of EGC (*H. pylori-*eradicated patient, invasion depth pT1a, and differentiated type adenocarcinoma) with improved visibility by TXI compared with WLI is shown in Fig. 4 (TXI: 3 [2–4], and WLI: 2 [1–4]).

## Discussion

To the best of our knowledge, this is the first study to compare the color differences (the CIE L*a*b* color space) and subjective visibility of EGCs by WLI and TXI mode 2 using the third-generation high-definition UTE and a new processor. The combination of WLI and UTE is widely used for screening EGD in clinics and health check-up institutions. TXI mode 2, without color enhancement, which provides similar images to WLI, might be useful for screening EGD using the currently available diagnostic methods. Therefore, in this study we exploratively investigated the effects of the combination of TXI mode 2 and UTE on the visibility of EGCs. In this study, we clarified the objective efficacy of TXI mode 2, which demonstrates greater color differences, to detect EGC than WLI, irrespective of *H. pylori* status, and macroscopic type and location of the EGC. In addition, an advantage was also observed using TXI mode 2 for subjective evaluation using visibility score, not only regarding evaluation by expert endoscopists but also by trainee endoscopists. Although there was no significant difference in subjective visibility of EGCs between WLI and TXI in *H. pylori*-positive patients, objective color differences evaluated by TXI mode 2 were significantly greater than that by WLI. Therefore, even if expert endoscopists select TXI mode 2 for endoscopic screening, attention should be paid, particularly to *H. pylori*-positive patients. As this study was a preliminary study with a small sample size, a study should be conducted in the future to investigate whether similar results can be obtained for various gastric cancer types (i.e., clinical stage, pathological differentiation, depth, and location), as a multicenter prospective study enrolling many patients.

Although TXI, which utilizes Retinex theory-based image processing technology, is reported to specifically enhance 3 imaging factors of WLI (texture, brightness, and color) to clearly define subtle tissue differences (e.g., normal mucosa and neoplasm), to our knowledge, there are only a few reports to date evaluating the efficacy of TXI for the diagnosis of EGC^9,10^. In a small study analyzing 12 EGCs imaged using a conventional endoscope (GIF-H290Z and GIF-EZ1500) and the EVIS XI system, Ishikawa et al.^9^ were unable to show significant color differences around the EGC between WLI and TXI mode 2 (8.0 ± 4.2 and 10.2 ± 8.4, respectively). In addition, Abe et al.^10^ were also unable to show the efficacy of TXI mode 2 (12.7 ± 6.1) for the evaluation of color differences around the EGC compared with WLI (10.3 ± 4.7) in 20 EGCs imaged using a conventional endoscope (GIF-H290Z) and the LUCERA ELITE system. Although the diagnostic efficacy of TXI determined in our study cannot be directly compared with those of previous reports, owing to the different study designs and samples, TXI mode 2 using the novel third-generation high-definition UTE with the EVIS XI system had greater color differences at 16.0 ± 10.1 than WLI. In general, improvement in the resolution, noise, and graduation of the endoscope and processor will be required to improve the detection rate of EGC. Therefore, we believe that combining the new third-generation UTE with the new processor increased the color differences around the EGCs, as well as the subjective visibility of the EGCs. In fact, we previously reported that color differences surrounding the atrophy produced by NBI using the GIF-1200N endoscope were significantly greater than those using GIF-290N (19.2 ± 8.5 vs. 14.4 ± 6.2, *P* = 0.001)^25^. Because TXI mode 2 using the third-genaration high-definition UTE with the EVIS XI system had greater color contrast than WLI, TXI mode 2 in combination with GIF-1200N and the EVIS XI system may be favorable option for screening endoscopy at health check-ups.

UTE is often performed transnasally to reduce invasiveness and patient distress, and because it does not require any sedation^26,27^. In fact, UTE without sedation saves costs for sedation, while providing similar patient satisfaction to conventional oral endoscopy with sedation^28^. Therefore, UTE is widely used for screening EGD in clinics and health check-up institutions in Japan. However, the major and serious disadvantages of first- and second-generation UTE that were used for screening include poor image quality and lower diagnostic accuracy of EGC^29^. It was therefore difficult to distinguish different structures and contrast of color between the EGC and background gastric mucosa, but the recently developed high-definition third-generation UTE has markedly improved image quality compared with standard-definition second-generation UTE and oral endoscopy^15–19,25^. Sugita et al.^18^ reported that the ability of third-generation UTE to detect EGC was significantly higher than that of second-generation UTE (accuracy: 80.8% vs. 71.6%, *P* = 0.017; sensitivity: 94.9% vs. 76.5%, *P* < 0.001; positive predictive value, 76.2% vs. 55.3%, *P* < 0.001; and negative predictive value, 94.1% vs. 73.5%, *P* < 0.001). A strength of this study is that we showed the efficacy of third-generation UTE using TXI mode 2 to evaluate color differences (the CIE L*a*b* color space) and subjective visibility between EGC and the non-neoplastic mucosa. Therefore, we believe that third-generation UTE with IEE methods, such as TXI mode 2, may be useful for identifying patients with gastric cancer at health check-ups through not only improved image quality, resolution, noise, and graduation, but also increased color differences.

This study has some limitations. First, this was a small-scale retrospective single-center study. Second, among the 111 EGC patients who were considered for inclusion, only 31 EGC patients were finally enrolled. In addition, only 2 images per EGC (1 WLI image and 1 TXI image) were evaluated regarding color differences and visibility scores, and hence there may be selection bias. Third, almost all of the EGC patients included in this study had differentiated-type adenocarcinoma with an invasion depth of pT1a. Therefore, the visibility of undifferentiated adenocarcinoma and submucosal invasive EGC using WLI and TXI mode 2 with high-definition UTE remains unclear. Fourth, because we enrolled patients scheduled for ESD, we had no data to directly evaluate the efficacy of detection of EGC in actual clinical practice and health check-ups.

In conclusion, TXI mode 2 using the novel high-definition UTE effectively improved objective and subjective visibility of EGCs compared with WLI. Our results suggest that TXI mode 2 using the novel high-definition UTE might contribute to the detection of EGC in daily clinical practice. A further large-scale prospective study is warranted to validate the real-time detection ability of EGCs during screening EGD.

## Data Availability

The datasets generated and/or analyzed in this study are available from the corresponding author on reasonable request.
